# Ohmic Heating Extraction at Different Times, Temperatures, Voltages, and Frequencies: A New Energy-Saving Technique for Pineapple Core Valorization

**DOI:** 10.3390/foods11142015

**Published:** 2022-07-07

**Authors:** Mohsen Gavahian, Rachael Chu

**Affiliations:** Department of Food Science, National Pingtung University of Science and Technology, Neipu, Pingtung 91201, Taiwan; rachael.r.chu@gmail.com

**Keywords:** emerging technologies, pineapple core, ohmic heating extraction, energy consumption, valorization, *Ananas comosus*

## Abstract

Pineapple core is considered a processing by-product. This study proposed and evaluated an ohmic heating extraction-based valorization platform to obtain value-added bioactive compounds from pineapple core and studied the effects of four important processing parameters. In this sense, a Taguchi design (L_16_(4)^4^) was used to assess the effects of temperature (70, 80, 90, and 100 °C), time (15, 30, 45, and 60 min), voltage (110, 160, 210, and 260 V), and frequency (60, 340, 620, and 900 Hz) on heating rate, come-up time, energy consumption, system performance efficiency, total phenolic compounds (TPC), DPPH, and ABTS. Finally, a side-by-side comparison of optimized ohmic heating (OOH) and conventional extraction was performed, and chemical composition was compared by ultra-performance liquid chromatography equipped with photodiode array detection-mass (UPLC-DAD-ESI-MS-MS). According to the results, increasing temperatures enhanced system performance efficiency but negatively affected TPC and antioxidant values above 90 ℃. Similarly, prolonging the extraction (>30 min) decreased TPC. Further, increasing voltage (from 110 to 260 V) shortened the come-up time (from 35.75 to 5.16 min) and increased the heating rate (from 2.71 to 18.80 °C/min^−1^). The optimal conditions were 30 min of extraction at 80 °C, 160 V, and 900 Hz. Verification of the optimal conditions revealed that OOH yielded an extract with valuable bioactive compounds and saved 50% of the time and 80% of energy compared to the conventional treatment. The UPLC-DAD-ESI-MS-MS showed that there were similarities between the chemical profiles of the extracts obtained by conventional and OOH methods, while the concentration of major compounds varied depending on the extraction method. This information can help achieve sustainable development goals (SDGs) by maximizing the yield and minimizing energy and time consumption.

## 1. Introduction

Pineapple (*Ananas comosus*) is the third major tropical fruit with approximately 37 Mt global production [1,2]. It is also one of the significant commercial fruits of Taiwan, with an annual production rate of above 400,000 tons [3]. In addition to fresh consumption, this popular fruit has been used in the food processing industry for several processes, including canning. Accompanied by high production rates, vast amounts of by-products from various parts of the pineapple, including pineapple cores, are generated. It has been indicated that dealing with pineapple processing by-products, which are more than 60% of its total weight, is a critical environmental and economic concern [4]. While being a burden for the industry to be handled after production, these by-products contain valuable bioactive compounds such as phenolic compounds [5,6,7]. These compounds are secondary plant metabolites with health benefits and therapeutic properties, including anti-inflammatory, anticancer, and antithrombotic effects [8]. Therefore, researchers are working on extracting these compounds from fruit processing by-products. 

Among extraction technologies, traditional thermal technology, which is based on the principle of heat transfer through a heating medium, has been utilized widely. However, it has several drawbacks, such as high energy consumption and the degradation of bioactive compounds due to the long processing time. Hence, volumetric heating technologies, such as ohmic heating, have emerged to overcome the drawbacks of traditional extraction. According to the Joules law, ohmic heating is based on the conversion of an electrical current to heat due to the electrical resistance of the sample. The volumetric heat generated may provide several benefits, including a short processing time and lower energy consumption, while it is also possible to take advantage of its possible non-thermal effects. [9,10].

Despite the simple design, ohmic processing involves several processing variables, such as temperature, time, electric field, voltage, current, and frequency, with different effects on the extraction process and physicochemical properties of the extracts. Recently published data indicated that using suitable ohmic heating processing conditions is beneficial in boosting extraction, and examples include the reports on extracting anthocyanins from grape skin [11], bioactive compounds from color potato [12], phenolics from rambutan peels [13], bioactive compounds from tomato by-products [14], and phenolics from vine-pruning residue [15]. However, there is limited information on pineapple core extraction through ohmic heating, and it is unclear how the processing parameters may affect the properties of pineapple core extract. Therefore, the present study aims to evaluate the effects of ohmic extraction conditions, including temperature, time, voltage, and frequency, on the energy consumption, system performance efficiency, and chemical characteristics of the extract. 

## 2. Materials and Methods

### 2.1. Chemical Reagents

Gallic acid monohydrate, 2-tert-Butyl-4-hydroxyanisole (BHA), 2,2-diphenyl-1-picrylhydrazyl (DPPH), and L-Ascorbic acid were purchased from Sigma-Aldrich, USA. Potassium permanganate and Folin-Ciocalteu’s reagent were collected from Merck, Germany. Sodium carbonate and methanol were obtained from Alfa Aesar and Macron Fine Chemicals™, USA, respectively.

### 2.2. Sample Preparation

Fresh golden-diamond (Tainung-17) pineapple fruit (*Ananas comosus*) was obtained from a farm in Pingtung County in Taiwan. The pineapple core was collected and cut into 1×1×3 cm^3^ cylindrical shapes of length, width, and height using a commercial pineapple cutting tool. The sample was divided into 166.67 ± 1.00 g and packaged in airtight Ziploc packages. Then, stored at −20 °C (Caravell Tropicalized, Roskilde, Denmark) after a quick-freezing process (TSX40086D, Thermo Scientific, Asheville, NC, USA). These samples were used for further extraction after defrosting to refrigeration temperature. 

### 2.3. Ohmic System and Extraction Method

**Figure 1:**
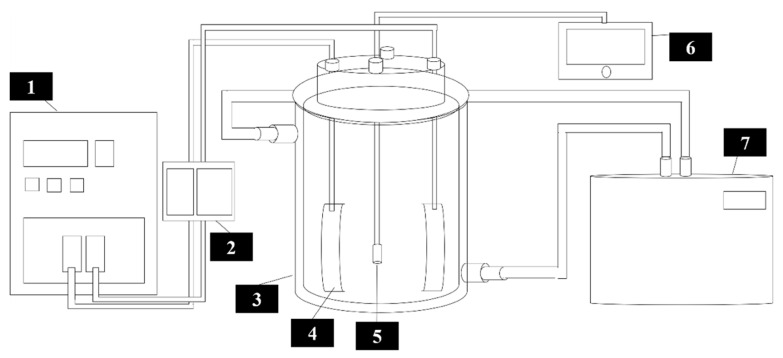
A schematic representation of the ohmic heating extraction-based valorization system designed and developed in the emerging food processing laboratory of the National Pingtung University of Science and Technology. 1: Power supply equipped with online monitoring system. 2: Automatic monitoring and recording device. 3: Extraction chamber. 4: Ohmic electrode. 5: K-type thermocouples. 6: Twelve-channel temperature monitoring system. 7: Temperature regulating system for the conventional heating treatment.

An ohmic heating system was used for the extraction process with a maximum voltage and frequency of 300 V and 999.9 Hz, respectively (SPA-1105B, Satech power, New Taipei City, Taiwan). The system consists of two stainless steel electrodes (32 × 10^−4^ m^2^) with a 9.0 cm gap and a cylindrical double-layer glass chamber with a capacity of 1 L. A recording system and a twelve-channel temperature monitoring device (JTT2012, Jianc, New Taipei City, Taiwan) were used with K-type thermocouples (K-2M, Jianc, New Taipei City, Taiwan) to save the data every 1 s (Figure 1). Processing conditions were selected based on the preliminary results reported on an ohmic system design and development [16] and the preliminary data on the pineapple core extraction at a single voltage of 110V (Unpublished data, abstract is submitted to 21st IUFoST World Congress 2022). Briefly, 500 g of the mixture of water and pineapple core samples with a ratio of 2:1 was used. Higher ratios yielded a diluted extract with difficulties in measuring the bioactive compounds, while lower ratios resulted in inadequate extraction due to a lack of sufficient contact/immersion between the extraction solvent and all the samples. 

### 2.4. Engineering Parameters 

#### 2.4.1. Come-Up Time, Heating Rate, and EF

Based on the recorded time, energy, and temperature data, the heating rate and electrical field were calculated through the method suggested in the literature [17] (Equations (1) and (2)):

(1) $$\mathrm{HR}=\frac{∆T}{∆t}$$

where the HR is the heating rate (°C/min), ∆T is the temperature change (°C), and ∆t is the time consumption (min) for reaching the come-up time; and

(2) $$\mathrm{EFS}=\frac{V}{\mathrm{EG}}$$

wherein EFS, V, and EG are present as the electrical field (V/cm), voltage (V), and electrical gap (cm), respectively.

#### 2.4.2. Electrical Energy Consumption (EEC)

The calculation of EEC was completed using Equation (3) [18]:

(3) $$\mathrm{EEC}=\sum_{t_{0}}^{t_{1}}E$$

wherein the EEC is the electrical energy consumption (Wh) during the total amount of energy output in the unit of time; t_0_ and t_1_ are the initial and final time of heating (hour), respectively; and E is energy taken during the time (Wh). 

#### 2.4.3. System Performance Efficiency (SPE)

SPE was calculated as specified in Equation (4) [19]:

(4) $$\mathrm{SPE}=\frac{C_{p}\times ∆T\times W}{\mathrm{EEC}}\times 100\%$$

where the C_p_ is the special heat of the sample (kJ/kg.°C), ∆T is the temperature changed to the targeted temperature (°C), W is the total weight of sample (kg), and EEC is the electrical energy consumption (Wh) required to reach the targeted temperature.

### 2.5. Chemical Assessment of Extracts

#### 2.5.1. Total Phenolic Content (TPC) 

According to the Folin-Ciocalteu assay in the literature [16], filtrated extracts or standards with different concentrations were added at 25 μL with 1 M sodium carbonate 125 μL through a 100 μL Folin–Ciocalteau reagent. The mixture was assessed by spectrophotometer (Thermo Scientific 1510, Vantaa, Finland) after being incubated at an ambient temperature in the dark for 30 min, and then detected at 765 nm.

#### 2.5.2. DPPH (2,2-diphenyl-1-picrylhydrazyl) Assay

The antioxidant activity was detected by the DPPH method, following a protocol explained in the literature [20]. Briefly, the 0.1 mM DPPH was dissolved in methanol and mixed with the extracts. The mixture was left in a dark place for 30 min, and then analyzed by a spectrophotometer at 517 nm.

#### 2.5.3. ABTS (2,20-azino-bis (3-ethylbenzothiazoline-6-sulphonic acid)) Assay

In line with a method described in a previous report [21], the mixture generated the ABTS agent through 2.45 mM potassium permanganate and 7.00 mM ABTS aqueous water solution. After the ABTS agent was kept in the dark for 12–16 h at room temperature, it was diluted to 0.70 ± 0.02 nm at 734 nm. Briefly, the different diluted extracts (100 μL) were mixed with 100 μL diluted ABTS agent and measured at 734 nm by spectrophotometer after being kept in the dark for 6 min. 

### 2.6. Taguchi Method 

The experimental design was performed based on the Taguchi approach to evaluate the influence of independent parameters, including extraction temperature (70, 80, 90, and 100 °C), time (15, 30, 45, and 60 min), voltage (110, 160, 210, and 260 V), and frequency (60, 340, 620, and 900 Hz). After the extraction process, the extracts were filtered and stored at −20 °C until analysis.

#### 2.6.1. Treatments in the Study Design 

In the present study, L_16_(4)^4^ orthogonal arrays with four differing parameters and four levels were used in the Taguchi method (Table 1), similar to the approach reported in a recently published manuscript, but with small modifications [22]. 

#### 2.6.2. Analyzing the Effects of the Processing Parameters and Optimal Conditions

To evaluate the effects of the processing parameters, the higher the signal-to-noise ratios (*S/N*), the better the heating rate, system performance efficiency, and total phenolic content were considered. In contrast, the concept of the lower *S/N*, the better was considered for a come-up time, energy consumption during the come-up time, electrical energy consumption, and antioxidant assays results. The calculation of the *S/N* ratio was performed according to [23], as explained in Equations (5) and (6), respectively:

(5) $${\frac{S}{N}}_{\mathrm{higher}}=-10\log\left(\frac{1}{n}\overset{n}{\underset{i=1}{\sum }}\frac{1}{y_{1}^{2}}\right)$$

(6) $${\frac{S}{N}}_{\mathrm{lower}}=-10\log\left(\frac{1}{n}\overset{n}{\underset{i=1}{\sum }}y_{1}^{2}\right)$$

where n and y_1_ are the numbers of experiment repetitions and the values of repetitions, resulting in the same condition. 

Based on the result of S/N, the OH system was run at the optimal conditions. The extracts’ chemical compositions and system performances at optimal ohmic heating (OOH) were then compared with that of the conventional heating (CH) method. The CH was performed using the same extraction chamber as the ohmic system, along with a hot-oil-bath-based heating system at 100 °C for 60 min. The heat transfer medium was silicon oil (Silicone Fluid 100 CS, XIAMETER™ PMX-200, Charleston, IL, USA). The processing parameters were monitored and recorded using the same devices employed for the ohmic system, along with an oil batch online monitoring system. 

### 2.7. Identification by UPLC-Q-Exactive Plus Orbitrap MS/MS Analysis

The extracts obtained at the optimal conditions of the ohmic process were analyzed and compared with that of the conventional method through ultra-performance liquid chromatography equipped with photodiode array detection-mass (UPLC-DAD-ESI-MS-MS, a combination of liquid chromatography and mass spectroscopy. 

#### 2.7.1. Liquid Chromatography

The phenolic compounds of the pineapple core extracts were analyzed by ultra-performance liquid chromatography (UPLC) (Ultimate 3000 UHPLC System, Thermo Fisher Scientific, USA) controlled by Thermo Xcalibur software (Thermo Fisher Scientific, USA) according to a previously utilized method [24]. The separation was boosted using a reversed-phase 130 Å, 2.1 mm × 100 mm, 1.7 um C18 column (RP-18 column, Waters^TM^, Milford, MA, USA). The A and B mobile phases consisted of 2% acetonitrile-0.1% formic acid and 99.9% acetonitrile-0.1% formic acid, with a continuous flow rate kept at 0.25 mL/min, and the gradient program was: 0% B at 2 min, 0–20% at 2–5 min, 20–20% at 5–7 min, 20–35% at 7–10 min, 35–35% at 10–12 min, 35–85% at 12–15 min, 85–85% at 15–17 min, 85–0% at 17–20 min, and 0–0% at 20–25 min. The sample injection volume was 5 µL and the column temperature was kept constant at 40 °C. 

#### 2.7.2. Mass Spectrometry

The Quadrupole-Orbitrap mass spectrometer (Q-Exactive Plus™, Thermo Fisher Scientific, United States) was performed in the negative ion mode. The scan mass range was set at *m/z* 100–900 with a fragment resolution of 70,000 FWHM, applying the ESI (−) model at 3.6 kV. The accuracy error threshold was constant at 5 ppm. The capillary temperature kept at 320 ℃ and collision-induced dissociation (CID) was continued at 30 V.

### 2.8. Statistical Analysis 

All the experiments were repeated three times. The data were reported as mean values of triplications, and the significance of differences was determined by Duncan’s test at a level of 5% using the ANOVA function of SPSS (IBM SPSS Statistics, SPSS, Inc., Chicago, IL, USA).

## 3. Results and Discussion

### 3.1. Come-Up Time, Heating Rate, and Electrical Parameters 

**Figure 2:**
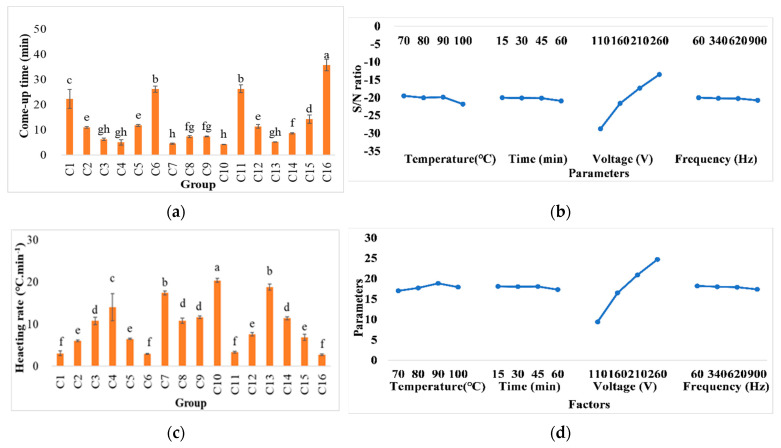
Comparisons of come-up time (**a**) and response graph of lower-better (**b**) and heating rate (**c**), as well as the values with higher-better (**d**) signal-to-noise ratios of different conditions in ohmic heating treatments. Values C1–C16 represent pineapple cores in different treatment conditions including temperature (°C), time (min), voltage (V), and frequency (Hz), respectively: 1: 70, 15, 110, and 60; 2: 70, 30, 160, and 340; 3: 70, 45, 210, and 620; 4: 70, 60, 260, and 900; 5: 80, 15, 160, and 620; 6: 80, 30, 110, and 900; 7: 80, 45, 260, and 60; 8: 80, 60, 210, and 340; 9: 90, 15, 210, and 900; 10: 90, 30, 260, and 620; 11: 90, 45, 110, and 340; 12: 90, 60, 160, and 60; 13: 100, 15, 260, and 340; 14: 100, 30, 210, and 60; 15: 100, 45, 160, and 900; and 16: 100, 60, 110, and 620. The same letters show insignificant differences in the same rows (*p* < 0.05). The results are reported as means ± SD.

The come-up time and heating rate in various extraction conditions are shown in Figure 2. It was observed that groups C1, C6, C11, and C16 showed the higher come-up times and lowest values of the heating rates. Based on the response graph of S/N in the Taguchi method, the voltage was the most influential parameter in terms of heat generation. This is because an increase in voltage promotes the movement of electrons, resulting in higher heating rates [25]. According to Figure 2, other independent parameters were less influential than voltage. This observation was in line with those reported in the literature. For example, a recent study [9] showed that increasing the electric field strengths from 4.28 to 15.71 V/cm reduced the come-up time from 12 to 4 min and increased the heating rate from 5.83 to 17.50 °C/min in the ohmic extraction of wheat bran. Another study on the ohmic concentration of orange juice [26] mentioned that processing time was reduced from 98.33 ± 4.21 to 40.86 ± 6.53 min when the voltage gradient increased from 15 to 30 V/cm.

Regarding the heating rate (Figure 2), the voltage mainly affects the rising temperature. In addition to the higher input of electrical power at higher voltages, it can be hypothesized that cell membrane stability can be disrupted at higher values of electrode movement. The electrolyte released from cells due to cell membranes being damaged by high electrical fields can increase the electrical conductivity, enhancing the heating efficiency. Meanwhile, the higher extraction yields and antioxidant values can be hypothetically explained by higher heating rates, higher rates of cell disruption, and possible electro-permeabilization and electroporation at high voltage conditions. In line with these observations, a study on the ohmic extraction of orange pectin documented that yield and electrical conductivity were increased by increasing the input voltage from 30 to 100 V. Therefore, the electrical current is beneficial for increasing the cell structure damage and diffusion rate [27].

The variations in processing parameters, including the electrical field, current, and power measured at constant voltage, to reach the target temperature are presented in Table 2. As exposed to the electric field, charged ion collisions continually happened while attracted by the electrodes as the providers of the opposite charges, increasing temperature. Further, the higher energy existing at high temperatures promotes electron movements, i.e., higher electrical current values are expected at higher electrical powers. Meanwhile, an increase in the frictional collision between the molecules may increase apparent resistance by considering the possibility of the leakage of plant cell ingredients [25,28]. A similar result appeared when phenolic compounds were extracted from wheat bran, showing that different voltage gradients (44 and 14 V/cm) can considerably enhance the heating rate and efficiency [29]. Moreover, a previous study on infant formula processing mentioned that the heat efficiency depends on the voltage gradient [30]. Similar observations were reported in the case of red beetroot and banana pulp treatment. A short time was needed to observe bubbles from the electrodes at high voltages, which can be considered a sign of electrochemical reactions in ohmic heating. This is related to effective heat generation and higher input power at higher voltages, promoting electrochemical reactions and electrode corrosion [31]. It was reported that electrolysis and metal corrosion were exacerbated by high voltage [32]. These may limit the use of very high voltages in commercial ohmic units for pineapple core valorization and should be considered in the system and process designs for industrial implementation. Furthermore, such electrochemical reactions on bioactive compounds should be considered, as further discussed in this paper in a section on TPC. 

### 3.2. Energy Consumption during Extraction

**Figure 3:**
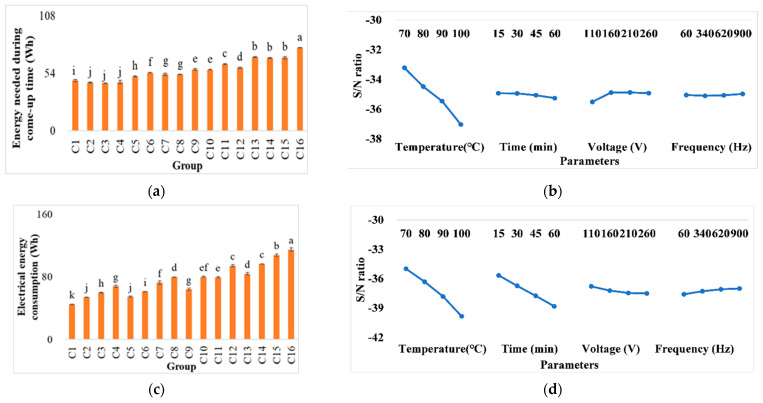
(**a**) The energy needed during come-up time, (**b**) response graph, (**c**) electrical energy consumption, and (**d**) response graph of the lower-better signal-to-noise ratios of the different conditions in ohmic heating. Values C1–C16 represent pineapple cores in different treatment conditions including temperature (°C), time (min), voltage (V), and frequency (Hz), respectively: 1: 70, 15, 110, and 60; 2: 70, 30, 160, and 340; 3: 70, 45, 210, and 620; 4: 70, 60, 260, and 900; 5: 80, 15, 160, and 620; 6: 80, 30, 110, and 900; 7: 80, 45, 260, and 60; 8: 80, 60, 210, and 340; 9: 90, 15, 210, and 900; 10: 90, 30, 260, and 620; 11: 90, 45, 110, and 340; 12: 90, 60, 160, and 60; 13: 100, 15, 260, and 340; 14: 100, 30, 210, and 60; 15: 100, 45, 160, and 900; and 16: 100, 60, 110, and 620. The same letters show insignificant differences in the same rows (*p* < 0.05). The results are reported as means ± SD.

The electrical energy consumption at various extraction conditions is illustrated in Figure 3. Among the treatments, C16 has the highest energy consumption (114.94 ± 2.36 Wh), while C1 has the lowest energy consumption (44.99 ± 0.28 Wh). The difference between the highest and lowest values was about 61%, meaning that the optimal conditions can save significant energy consumption and processing costs. Further, about 42% more energy was used during the come-up time when the target temperature was changed by 30 °C (from 70 to 100 °C). The data showed that extraction temperature is the most critical parameter that affects energy consumption, which was the case during both come-up time and total processing time, i.e., energy consumption during the come-up time and total electrical energy consumption, respectively. 

Higher voltages reduced the energy consumption required to heat the sample. Conversely, lower voltages increased the required electrical energy consumption. The reduced energy consumption during the come-up time was because of the effects of high voltage on reducing come-up time, reducing the amount of energy loss to the surrounding area. Similarly, an investigation on the ohmic processing of infant food showed that 24 V/cm has a higher heat efficiency than 8 V/cm, reducing the come-up time and energy consumption from 39 to 4 min and 26 to 15 Wh, respectively [30].

Conversely, another study [33] demonstrated that the system performance coefficient was promoted from 0.764 to 0.939 when the electrical field was changed from 55 to 30 V/cm for pomegranate juice processing. A similar trend was reported in another paper on mulberry juice concentration, with a positive coloration between energy consumption and voltage increase. In the studies mentioned above, the evaporation rate was promoted by increasing the voltage gradient, which increased the ion concentration and solution electrical conductivity, resulting in different observations from the present study. Overall, it can be considered an advantage of ohmic heating that volumetric heat generation can significantly boost the heating rate and reduce the processing time [34,35,36]. 

### 3.3. System Performance Efficiency 

**Figure 4:**
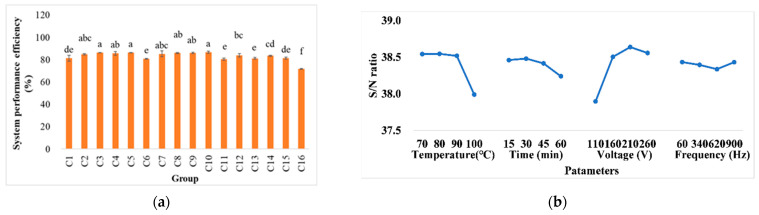
(**a**) The system performance efficiency, and (**b**) response graph of higher-better signal-to-noise ratios of various conditions in ohmic heating. Values C1–C16 represent pineapple cores in different treatment conditions including temperature (°C), time (min), voltage (V), and frequency (Hz), respectively: 1: 70, 15, 110, and 60; 2: 70, 30, 160, and 340; 3: 70, 45, 210, and 620; 4: 70, 60, 260, and 900; 5: 80, 15, 160, and 620; 6: 80, 30, 110, and 900; 7: 80, 45, 260, and 60; 8: 80, 60, 210, and 340; 9: 90, 15, 210, and 900; 10: 90, 30, 260, and 620; 11: 90, 45, 110, and 340; 12: 90, 60, 160, and 60; 13: 100, 15, 260, and 340; 14: 100, 30, 210, and 60; 15: 100, 45, 160, and 900; and 16: 100, 60, 110, and 620. The same letters show insignificant differences in the same rows (*p* < 0.05). The results are reported as means ± SD.

Figure 4 shows the system performance efficiency at various conditions in ohmic heating. It indicates that the temperature and voltage applied are the significant factors that affect the SPE. It is worth highlighting that at temperatures close to 100 °C, there is a significant adverse effect on system performance. According to the literature [36], the possible cause is the formation of bubbles and the loss of water when the boiling point is reached. The bubbling phenomenon may cause localized changes in the electrical conductivity, making the electric field uneven and bumpy and resulting in local temperature differences [37].

On the other hand, it occurs from the hydrogen bubbles due to the electrolysis reaction of corrosion, which is promoted at acidic and high voltage conditions [38]. The high temperature increases electrical conductivity, which provides greater current flow and arouses the non-conductive bubbling, especially at a higher voltage [32]. At the same time, as a previous study [36] indicated, voltage increased the benefits from decreasing energy efficiency loss. The values of improvement potential from 67.07 to 85.50% increased the voltage gradient from 6 to 16 V/cm in tomato drying. The results of an investigation on the ohmic heating extraction of red beetroot showed that energy efficiency was increased as voltage increased [39]. According to a study on the ohmic cooking of noodles, heat loss was found at lower electric field conditions. A previous study indicated that more heat is lost to the environment at longer heating times [40]. According to another study, the value of system performance coefficient was increased from 0.46 ± 0.02 to 0.58 ± 0.02 by changing the treatment conditions from 10 to 17.50 V/cm [19]. Conversely, there may be significant divergent trends based on food characteristics if the treatments affect electrical conductivity (e.g., the evaporation process). In a study on the inactivation of pathogenic bacteria in various degrees of the Brix of apple juices, the highest system performance efficiency was observed at 30 V/cm, which was more than those observed at 40, 50, and 60 V/cm for apple juice over 24° Brix [41]. Nevertheless, the trend in voltage is still seen as the most important factor affecting system performance, and the value increased along with higher applied voltage.

### 3.4. Extraction Yield of TPC

**Figure 5:**
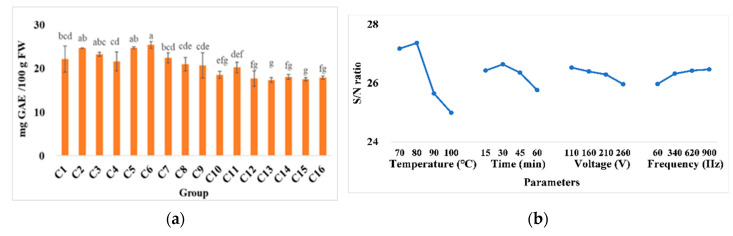
(**a**) The total phenolic content, and (**b**) response graph of higher-better signal-to-noise ratio of various conditions in ohmic heating. Values C1–C16 represent pineapple cores in different treatment conditions including temperature (°C), time (min), voltage (V), and frequency (Hz), respectively: 1: 70, 15, 110, and 60; 2: 70, 30, 160, and 340; 3: 70, 45, 210, and 620; 4: 70, 60, 260, and 900; 5: 80, 15, 160, and 620; 6: 80, 30, 110, and 900; 7: 80, 45, 260, and 60; 8: 80, 60, 210, and 340; 9: 90, 15, 210, and 900; 10: 90, 30, 260, and 620; 11: 90, 45, 110, and 340; 12: 90, 60, 160, and 60; 13: 100, 15, 260, and 340; 14: 100, 30, 210, and 60; 15: 100, 45, 160, and 900; and 16: 100, 60, 110, and 620. The same letters show insignificant differences in the same rows (*p* < 0.05). The results are reported as means ± SD.

Based on the results presented in Figure 5, four factors affect TPC, especially temperature, which has a significant downtrend from left to right. It likely shows that the phenolic compounds deteriorated when the extraction temperature was over 80 °C and the processing time was over 30 min. This may be attributed to the thermal damage to these thermolabile compounds. A similar result was found in studies conducted by other researchers [42], where TPC degradation happened as extraction time increased at extraction temperatures above 80 °C at 220 V. This result was also supported by observations in a previous study on orange concentration, where a greater degradation was reported at processing conditions that involved higher temperatures [43].

The electric field increase at the elevated applied voltage likely enhances the degradation of the phenolic compounds. Even with the benefit of phytochemicals released from the unstable membrane due to an oscillating electric field in ohmic heating, the degradation also may occur considering the chemical structure of the phytochemicals. Researchers [44] have also observed a similar tendency in TPC loss in the thermal treatment of fresh pineapple juice. The research found a higher TPC loss (25.2–31.7%) at higher electrical fields (28–36 V/cm) in comparison with 6.710.4% lost at lower electrical fields (16–24 V/cm). The development of TPC loss is inversely proportional to the electric field increase from 16 to 36 V/cm. According to literature [45], such an increase in degradation was caused by the increasing collision force between the molecules. However, the opposite TPC extraction trend was shown in previous research [16]. The results of the Taguchi method revealed that there is a positive correlation between voltage and TPC. The different influences of the parameters on ohmic heating extraction are likely to be attributed to the properties of the samples [46]. Otherwise, higher frequency values reduced TPC loss. A study on whey acerola-flavored drink processing reported that the highest degradation of TPC value (45.66 ± 3.27 µg Gallic acid/g) was observed while using a lower voltage (60 V) and frequency (60 Hz) in ohmic heating. The results can be explained by the assumption that the polarization reduction by molecular orientational movements may decrease at high frequencies [47]. On the other hand, it should be noted that the electrochemical reactions may lead to oxidation of the phytochemicals by the electrolysis reactions and electrode corrosion at lower frequencies. A suitable frequency can enhance the extraction while minimize the degradation of extracted compounds. As an example, it was reported that during pomelo juice ohmic pasteurization, TPC increased by 10%, depending on the electrical frequency applied [25]. 

### 3.5. Radical Scavenging Assays 

**Figure 6:**
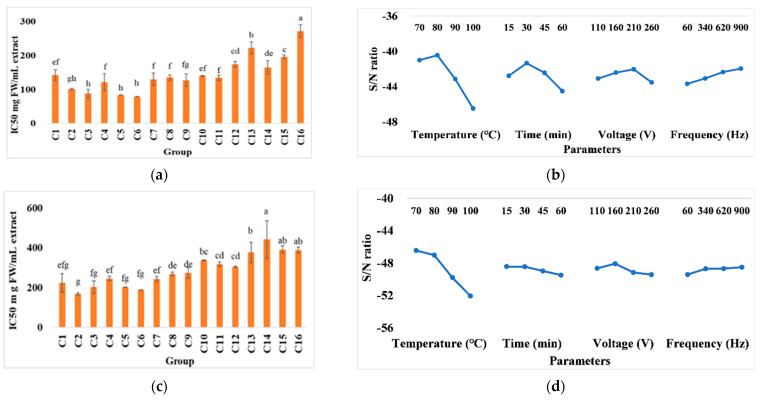
(**a**) DPPH assay with (**b**) the response graph of lower-better and (**c**) the ABTS assay with (**d**) the response graph of the lower-better signal-to-noise ratios of various conditions in ohmic heating. Values C1−C16 represent pineapple cores in different treatment conditions including temperature (°C), time (min), voltage (V), and frequency (Hz), respectively: 1: 70, 15, 110, and 60; 2: 70, 30, 160, and 340; 3: 70, 45, 210, and 620; 4: 70, 60, 260, and 900; 5: 80, 15, 160, and 620; 6: 80, 30, 110, and 900; 7: 80, 45, 260, and 60; 8: 80, 60, 210, and 340; 9: 90, 15, 210, and 900; 10: 90, 30, 260, and 620; 11: 90, 45, 110, and 340; 12: 90, 60, 160, and 60; 13: 100, 15, 260, and 340; 14: 100, 30, 210, and 60; 15: 100, 45, 160, and 900; and 16: 100, 60, 110, and 620. The same letters show insignificant differences in the same rows (*p* < 0.05). The results are reported as means ± SD.

Antioxidant activity was significantly impacted by extraction time, temperature, voltage, and frequency. According to Figure 6, C3, C5, and C6 showed the lowest values of IC50 in the DPPH assay. A similar result also was found in the ABTS assay. The trend is reflected in the response graph of the signal-to-noise ratios of DPPH and ABTS. The free radical scavenging ability decreased along with the rising extraction temperature. It shows the lowest value at 30 min, but it increased with a processing time of over 30 min, which is the same as the TPC trends. The related report encouraged a higher temperature for releasing the phenolic compounds from lignocellulosic materials [15], which is relevant considering that samples used in the present study were subjected to freezing and defrosting processes before extraction. Such a sample preparation may facilitate the release of bioactive compounds from damaged cell membranes. In addition, it can be understood from the results that the high extraction temperature over a long time may decrease the antioxidation ability through the degradation and oxidation of the phenolic compounds.

It is generally believed that an enhanced cell membrane permeability and electrochemical reaction may occur when an electrical current passes through the biomaterials. A study on whey acerola-flavored drink pasteurization showed that samples possess 8.88 ± 0.31 and 8.48 ± 0.37 µg Trolox Equation/g when treated at a voltage of 80 and 45 V, respectively [47]. Similarly, it was reported that the antioxidation capacity of grape juice concentrate increased from 77.0 to 85.3% as the voltage increased from 15 to 30 V/cm [48]. It was documented that thermal processing may promote the pro-oxidant and antioxidant molecules, along with antioxidant degradation and new antioxidant component generation [49]. Compared with the applied voltage of 210 V in the DPPH test, it has a better ability at 160 V in the ABTS test. Such different observations might be explained by considering the mechanisms involved in various antioxidant assays. The DPPH assay was determined under organic media, which is suitable for hydrophobic systems. On the other hand, the ABTS assay was determined under hydrophilic and lipophilic systems based on a one-electron antioxidant, which is soluble in aqueous and organic media [50]. Hence, the parameters have different effects on phenolic compounds with different chemical structures. Both tests show that the antioxidant activity increased by increasing frequency. As the antioxidant ability may increase by increasing the phenolic compounds, the oxidation of these compounds caused by the electrolysis reaction during ohmic heating extraction could be a reason for this observation [25]. At low frequencies, prolonged extraction times may provide suitable conditions for the electrode surface, ions, and electrolysis reaction. Therefore, using appropriate frequencies may reduce the immigration of metal ions from the electrodes to the sample [51]. 

### 3.6. Comparison between the Conventional System and Ohmic Heating at Optimal Conditions 

Based on “the lower-the-better” responses of the antioxidant characteristics in Figure 6, the second level of temperature (°C), the second level of time (min), the second level of voltage (V), and the fourth level of frequency (Hz) result in the minimum value of radical scavenging. Subsequently, the optimal factor level was in line with extraction conditions at 80 °C, 160 V, and 900 Hz for 30 min. The result of comparing conventional heating and optimal treatment with engineering and chemical performance is shown in Table 3. Spending half of the conventional extraction time and energy has 80.23% savings over conventional heating. Meanwhile, the TPC value in ohmic heating (26.61 ± 0.45 mg/100 g FW) performed significantly higher than in the conventional treatment (24.90 ± 0.06 mg/100 g FW). The antioxidant activity of the DPPH and ABTS assays also had the same trend. The ohmic heating treatments present in the DPPH and ABTS assays are 99.73 ± 14.31 and 189.68 ± 24.11 IC50 mg FW/mL extract, compared with conventional treatment at 125.15 ± 1.83 and 266.17 ± 0.84 IC50 mg FW/mL extract.

The results in Table 3 indicate that the value related to system performance under optimal conditions is superior to that of conventional heating, introducing the ohmic-assisted process as a time- and energy-saving alternative to the conventional method. 

**Figure 7:**
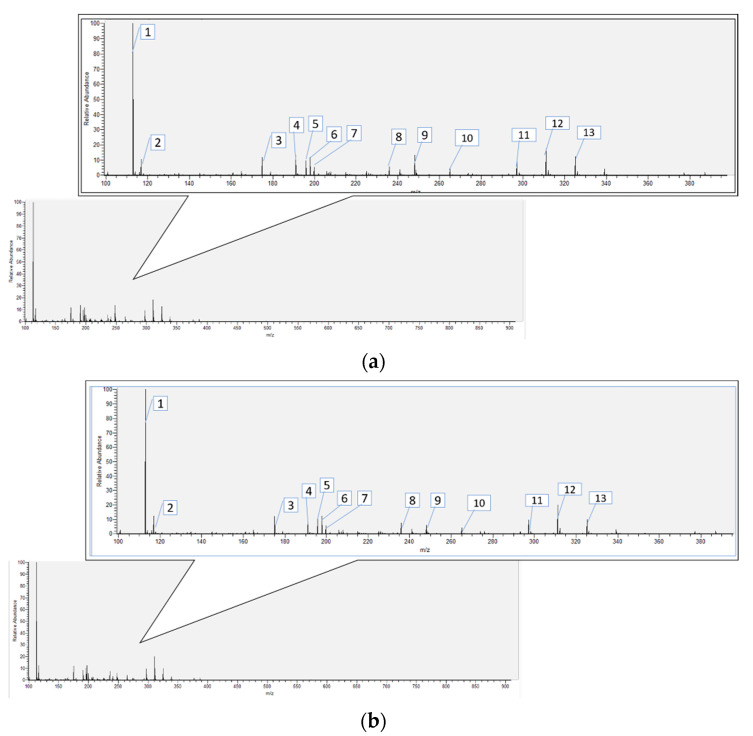
Mass spectrometry at a scan range of *m/z* 100–900 for [M + H]^−^ of the extracts obtained from optimal ohmic (**a**) and conventional heating (**b**).

Figure 7 presents the UPLC chromatograms of the extracts obtained by CH and OOH. The major components, according to the highest peak area, are specified in this Figure. Accordingly, a comparison between these major components in OOH and CH is presented in Table 4. The results show that both extracts have several peaks with a similar retention time (Rt) and with the same MS^n^ fragment, suggesting that there will be a general similarity between the chemical profiles of the extracts obtained by OOH and CH. However, a side-by-side comparison between these graphs revealed that peaks at the same Rt have different areas, which suggests a possible variation in the concentration of those components. Overall, the concentration of 13 major components (as examples) varied by between 4 and 40% (Table 4). For example, replacing CH with OOH increased the peak areas of compound No. 1 and compound No. 2 by 29.36 and 31.35%, respectively. While most of the major components showed a higher peak area in OOH sample, the peak area of compound No. 13 was 4.01% smaller than that of CH. While future in-depth studies may reveal the details of the effect of ohmic heating on the chemical composition of pineapple extract, it is expected that variations in the concentrations of the chemical profiles of the extracts could be related to the shorter process time and volumetric nature of ohmic heating, which can preserve thermolabile bioactive compounds. 

While further research may explore the details of the quantitative analysis of the chemical profiles of pineapple core extracts, some general information about the possible major components of pineapple core extracts is available in the literature, which may help with the interpretation of the qualitative results. For example, Mhatre et al. analyzed the extracts of a mixture of the core and pulp of pineapples by HPLC and observed that the significant components are ascorbic acid, flavon-3-ols, quercetin, and flavones [52]. A recent study analyzed pineapple extract by LC-ESI-UHR-QqTOF-MS and reported that caffeic, coumaric, and ferulic acid were the major compounds. Other studies have also identified p-hydroxybenzoic acid, 2,5-dihydroxybenzoic acid, and ferulic acid as polyphenols present in pineapple by-products [4]. This was similar to a previous UPLC-QToF MS-based analysis report [53] where the researchers pointed out that many of the major chemical components (e.g., caffeic, ferulic acid, and sinapyl derivatives) of pineapples belong to hydroxycinnamoyl glycosides. A similar hypothesis has been presented in other studies [24,53,54]. Meanwhile, other researchers have reported glutathione derivatives and the L-cysteine derivatives as the polyphenols of pineapples [53,54,55]. 

## 4. Conclusions

This study proposed a pineapple core valorization platform based on ohmic heating as an emerging thermal technology. This platform yielded an extract with considerable amounts of phenolic compounds and antioxidant activity. At the same time, the Taguchi approach revealed important data that should be considered for practical applications, by elaborating on the effects of processing parameters on the physicochemical properties of the extracts, as well as on energy consumption. The temperature and processing time were identified as the major influential parameters based on phenolic content and antioxidant capacity. The phenolic compounds were damaged more by inappropriate temperatures and extraction times than by voltage. Voltage was the most crucial factor that affected energy consumption. Voltage also had significant effects on heating efficiency and system performance. Therefore, in practice, increasing the voltage can save both energy consumption and processing time in such a platform. Besides, the effects of frequency on the chemical properties of extracts were more significant than on that engineering aspects of the process. The results indicated that understanding the effects of the processing parameters in ohmic heating-based valorization systems is crucial to enhancing process efficiency and achieving sustainable development goals (SDGs) as this can reduce energy consumption and maximize yield. Further, the freezing step can be excluded by installing an extraction system on-site (e.g., at a farm or pineapple processing factory) to further enhance the process sustainability and reduce the cost. In addition, the approach proposed in this study can reduce a pineapple’s processing by-product, usually regarded as waste, and provide further benefits to farmers and the food processing (e.g., canning) industry. 

## Figures and Tables

**Table 1:** The various experimental conditions as designed by the Taguchi method.

Group *	Temperature (°C)	Time (min)	Voltage (V)	Frequency (Hz)
C1	70	15	110	60
C2	70	30	160	340
C3	70	45	210	620
C4	70	60	260	900
C5	80	15	160	620
C6	80	30	110	900
C7	80	45	260	60
C8	80	60	210	340
C9	90	15	210	900
C10	90	30	260	620
C11	90	45	110	340
C12	90	60	160	60
C13	100	15	260	340
C14	100	30	210	60
C15	100	45	160	900
C16	100	60	110	620

* Values C1–C16 represent various conditions with various parameters, including temperature (°C), time (min), voltage (V), and frequency (Hz), respectively: 1: 70, 15, 110, and 60; 2: 70, 30, 160, and 340; 3: 70, 45, 210, and 620; 4: 70, 60, 260, and 900; 5: 80, 15, 160, and 620; 6: 80, 30, 110, and 900; 7: 80, 45, 260, and 60; 8: 80, 60, 210, and 340; 9: 90, 15, 210, and 900; 10: 90, 30, 260, and 620; 11: 90, 45, 110, and 340; 12: 90, 60, 160, and 60; 13: 100, 15, 260, and 340; 14: 100, 30, 210, and 60; 15: 100, 45, 160, and 900; and 16: 100, 60, 110, and 620.

**Table 2:** Changes in electrical values at target temperatures in pineapple core extraction in various ohmic heating conditions.

Group	EFS * (V/cm)	Current (A)	Power (kW)
C1	12.22 ± 0.00 ^d^	1.63 ± 0.00 ^j^	0.18 ± 0.00 ^g^
C2	17.78 ± 0.00 ^c^	2.68 ± 0.12 ^gh^	0.43 ± 0.02 ^f^
C3	23.33 ± 0.00 ^b^	3.60 ± 0.29 ^de^	0.76 ± 0.06 ^d^
C4	28.89 ± 0.00 ^a^	3.86 ± 0.99 ^cde^	1.01 ± 0.27 ^c^
C5	17.78 ± 0.00 ^c^	2.89 ± 0.05 ^fg^	0.46 ± 0.01 ^ef^
C6	12.22 ± 0.00 ^d^	2.00 ± 0.10 ^ij^	0.22 ± 0.01 ^g^
C7	28.89 ± 0.00 ^a^	4.96 ± 0.16 ^b^	1.30 ± 0.05 ^b^
C8	23.33 ± 0.00 ^b^	3.86 ± 0.22 ^cde^	0.81 ± 0.05 ^d^
C9	23.33 ± 0.00 ^b^	4.06 ± 0.18 ^cd^	0.85 ± 0.04 ^d^
C10	28.89 ± 0.00 ^a^	5.71 ± 0.05 ^a^	1.48 ± 0.01 ^a^
C11	12.22 ± 0.00 ^d^	2.38 ± 0.13 ^hi^	0.26 ± 0.01 ^g^
C12	17.78 ± 0.00 ^c^	3.56 ± 0.24 ^de^	0.57 ± 0.04 ^e^
C13	28.89 ± 0.00 ^a^	5.75 ± 0.04 ^a^	1.50 ± 0.01 ^a^
C14	23.33 ± 0.00 ^b^	4.19 ± 0.13 ^c^	0.88 ± 0.03 ^d^
C15	17.78 ± 0.00 ^c^	3.37 ± 0.25 ^ef^	0.54 ± 0.04 ^ef^
C16	12.22 ± 0.00 ^d^	2.14 ± 0.13 ^i^	0.24 ± 0.01 ^g^

The electrical values during different pineapple core extraction conditions. Values C1–C16 represent pineapple cores in different treatment conditions including temperature (°C), time (min), voltage (V), and frequency (Hz), respectively: 1: 70, 15, 110, and 60; 2: 70, 30, 160, and 340; 3: 70, 45, 210, and 620; 4: 70, 60, 260, and 900; 5: 80, 15, 160, and 620; 6: 80, 30, 110, and 900; 7: 80, 45, 260, and 60; 8: 80, 60, 210, and 340; 9: 90, 15, 210, and 900; 10: 90, 30, 260, and 620; 11: 90, 45, 110, and 340; 12: 90, 60, 160, and 60; 13: 100, 15, 260, and 340; 14: 100, 30, 210, and 60; 15: 100, 45, 160, and 900; and 16: 100, 60, 110, and 620. The same letters show insignificant differences in the same rows (*p* < 0.05). The results are reported as means ± SD. * Abbreviations: EFS, electric field strength.

**Table 3:** Comparison of optimization in ohmic heating and conventional heating.

Treatment	CT * (min)	HR (°C min)	CTEC (Wh)	EEC (Wh)	SPE (%)	TPC	DPPH	ABTS
OOH	9.54 ± 0.46 ^b^	7.80 ± 0.40 ^a^	48.58 ± 0.85 ^b^	61.48 ± 0.17 ^b^	88.70 ± 1.31 ^a^	26.61 ± 0.45 ^a^	99.73 ± 14.31 ^b^	189.68 ± 24.11 ^b^
CH	43.97 ± 0.64 ^a^	2.25 ± 0.02 ^b^	245.00 ± 5.00 ^a^	311.11 ± 11.11 ^a^	23.44 ± 0.34 ^b^	24.90 ± 0.06 ^b^	125.15 ± 1.83 ^a^	266.17 ± 0.84 ^a^

* OOH: optimal ohmic heating; CH: conventional heating; CT: come-up time; HR: heating rate; SPE: system performance efficiency (%); TPC: total phenolic content (mg/100 g fresh weight of pineapple core); DPPH: 2,2-diphenyl-1-picrylhydrazyl (IC50 mg FW/mL extract); ABTS: 2,2′-azino-bis(3-ethylbenzothiazoline-6-sulfonic acid) (IC50 mg FW/mL extract); CTEC: come-up time energy consumption; EEC: electrical energy consumption. The same letters show insignificant differences in the same columns (*p* < 0.05).

**Table 4:** A comparison of the major identified components present in pineapple core extracts from optimal ohmic heating and conventional heating.

No. *	Rt ** (min)	HR-ESI(−)-MS (*m/z*)	ESI(−)-MS^n^ Experiment (*m/z*)	Area Ratio *** (OOH/CH) (%)
1	1.2	113	69(100), 112(8)	129.36
2	16.9	117	118(5), 116(100)	131.35
3	10.4	175	175(17), 147(100), 119(4)	102.72
4	1.1	191	191(37), 173(5), 111(100), 87(24), 85(15)	139.97
5	20.2	196	196(100), 161(56)	122.57
6	20.1	198	198(100), 163(41), 161(16)	109.88
7	20.6	200	200(100), 165(27), 163(31)	107.05
8	20.7	236	191(17), 147(34), 124(7), 107(9), 106(7), 105(7), 104(100), 88(44)	107.04
9	17.7	265	265(100), 97(7)	134.39
10	17.9	297	298(7), 297(100)	127.47
11	19.2	311	312(8), 311(100)	109.7
12	15.8	325	326(7), 325(100)	127.96
13	17.6	339	340(6), 339(100)	95.99

* Compound numbers refer to the peaks specified in Figure 6. ** Rt: retention time; *** OOH: optimal ohmic heating; CH: conventional heating.

## Data Availability

The data presented in this study are available on reasonable request from the corresponding author.
